# The consequences of a compressed workweek: a systematic literature review

**DOI:** 10.1007/s00420-025-02153-8

**Published:** 2025-06-20

**Authors:** Vilde Bernstrøm, Daniele Alves, Inge Houkes, Andreas Lillebråten, Wendy Nilsen

**Affiliations:** 1 Work Research Institute, OsloMet, Oslo, Norway; 2 Department of Social Medicine, Care and Public Health Research Institute, Maastricht University, Maastricht, The Netherlands

**Keywords:** Extended shifts, Daily work hours, Compressed work schedule, Health

## Abstract

**Purpose:**

Compressed workweek arrangements, where employees work extended daily hours in exchange for fewer workdays, are adopted to address individual and organizational needs. While advocates highlight benefits such as improved work-life balance and reduced commuting time, the effects on employee health/well-being and work outcomes remain unclear. The objective of the current paper is to summarize existing knowledge on the longitudinal relationship between compressed workweeks and employee health/wellbeing and work outcomes.

**Methods:**

We conducted a systematic search in Medline, Embase, PsycINFO, Cinahl, and Web of Science in March 2023. We included peer-reviewed publications that empirically investigated the longitudinal relationship between compressed work schedules and employee health/well-being or work outcomes in employees working no more than 55 h a week. Twenty studies met the inclusion criteria. The study is registered in Prospero (CRD42020172595).

**Results:**

The 20 longitudinal studies yielded mixed results, identifying positive and negative effects on health/well-being and work outcomes. Most studies found no significant differences in at least one outcome. Results suggest that a compressed workweek increases sickness absence but also improves shift satisfaction. Predominantly negative health effects were observed when comparing a compressed workweek to fixed day work, mixed effects when comparing a compressed workweek with 12 h shifts to alternative shifts arrangements, and limited evidence for a compressed workweek with 10 h shifts.

**Conclusion:**

The impact of compressed workweeks remains uncertain, demonstrating mixed results on employee health and work outcomes. The findings vary depending on the length of shifts and alternative schedule.

## Introduction

Today a significant part of the work force works non-standard working time arrangements (Gracia et al. 2021). A compressed workweek is on such non-standard work hour arrangement used to address individual and organizational needs, such as increased work-life balance (Bambra et al. 2008), optimizing the use of personnel (Barnum 2011), or extending business hours (Wadsworth et al. 2010).

However, the consequences for employee well-being and for the quality of work remain unclear. It is crucial to understand the consequences of alternative work schedules so that employees and employers can adapt work hours to their needs safely.

In a compressed workweek, employees work extended daily work hours (≥ 10 h), also referred to as extended shifts, compensated by a reduced number of workdays so that the weekly work hours remain the same. For example, employees might work a compressed workweek of 10 h 4 days a week as an alternative to 8 h 5 days a week. A compressed workweek is advocated as a central tool in addressing individual, organizational and societal challenges. Proposed benefits include reduced travel time, improved work-life balance, optimizing the use of personnel, and for industries requiring 24-h service such as healthcare and law enforcement, reduced number of handovers, staffing shortages and increased continuity of care (Bambra et al. 2008; Dall’Ora et al. 2022; Ganong et al. 1976; Harris et al. 2015; Parkinson et al. 2018; Persson et al. 2006). However, the use of such schedules is highly debated; in countries where extended shifts are common, arguments are being put forth to revert to shorter shifts for the sake of employees' health and performance (Geiger-Brown and Trinkoff 2010; Harris et al. 2015).

Several literature reviews have supported negative health effects of extended daily work hours, including cardiovascular disease (Akira et al. 2014; Kang et al. 2012), musculoskeletal disorders (Bae and Fabry 2014; Banakhar 2017; Penso et al. 2022), metabolic syndrome (Akira et al. 2014), general health complaints (Bae and Fabry 2014) and accidents such as needlesticks injury (Bae and Fabry 2014; Imes et al. 2023). Most studies focus on shifts of 12 h or more, though some studies also find negative outcomes of shorter (e.g. 10 h) shifts (Bae and Fabry 2014). The adverse health outcomes associated with extended daily work hours have raised concerns about their effect on employees' work performance and safety, such as the consequences of increased fatigue on employees' cognitive skills (Bae and Fabry 2014). Indeed, studies have supported that working extended daily work hours is related to adverse job outcomes such as an increased risk of errors (Bae and Fabry 2014; Clendon et al. 2015; Dall'Ora et al. 2016; Leroyer et al. 2014). Notably, some literature reviews have found that the results on extended shifts are mixed and inconclusive, with results finding both positive and negative outcomes (Estabrooks et al. 2009; Harris et al. 2015).

Importantly, studies investigating the consequences of extended daily work hours often do not control for weekly work hours (Dall'Ora et al. 2016). Some employees who work extended daily work hours will also work extended weekly work hours (e.g., ≥ 10 h a day five days a week), either scheduled (Sallinen & Kecklund 2010; Wong et al. 2018) or because they work overtime on top of scheduled shorter shifts (Griffiths et al. 2014; Scott et al. 2006). A substantial part of the research on extended shifts are focused on profession such as health care workers and police officers. Yet, with an increasing demand for nursing care, some hospitals require or allow nurses to work extended shifts and an extended number of shifts per week—up to and in excess of 60 h per week (Surani et al. 2007). Similar patterns of extreme work hours are also reported from police offices (Vila 2006). Adverse health effects of working more than 50 or 55 h a week are well documented (Bonde et al. 2013; Kivimaki et al. 2015; Litwiller et al. 2017; Palmer et al. 2013; van Melick et al. 2014; Virtanen et al. 2015). A question is whether the adverse effects of extended daily workhours persists when total weekly work hours are kept constant and within safe limits.

Studies focusing specifically on compressed workweeks have found more optimistic results regarding employee health and performance (Bambra et al. 2008; Sallinen & Kecklund 2010), as compared to studies examining extended daily work hours in general. A 2007 systematic review on shift interventions, found inconsistent findings and insufficient evidence for definitive conclusions (Driscoll et al. 2007). Meanwhile, a 2008 systematic review on the health impacts of compressed workweeks was cautiously optimistic yet remained inconclusive (Bambra et al. 2008). The latter review indicated that compressed workweeks sometimes improved self-reported health and was seldom detrimental for employee health outcomes. Adverse organizational effects such as productivity and errors were small or absent. However, the studies had several methodological challenges such as small samples, inadequate control groups, only self-reported measures, and short follow-up times (Bambra et al. 2008). Therefore, the authors called for better designed studies to be able to gain more robust conclusions.

In sum, a compressed workweek might be a useful tool to address individual and organizational challenges, but their potential consequences are highly debated. As the former two reviews summarized, the evidence base of compressed workweek´s consequences up until 2005 (Bambra et al. 2008) and 2006 (Driscoll et al. 2007), there is a need for an updated systematic review examining studies conducted the past decade.

The objective of the present study is, therefore, to conduct a systematic literature review of longitudinal studies investigating the consequences of compressed, compared to non-compressed work for employee health, wellbeing, and work outcomes.

## Method

### Search process and study selection

**Fig. 1:**
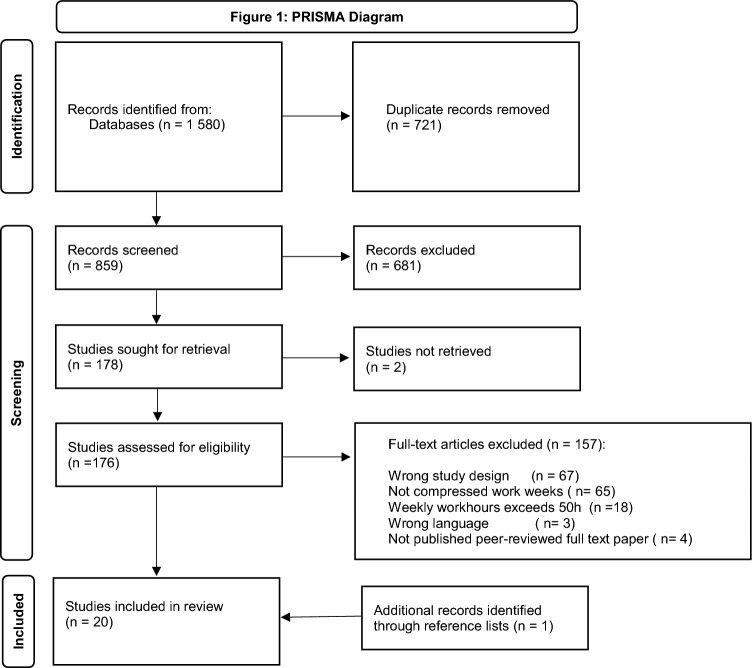
Flow chart of steps in systematic review

We conducted a systematic search of literature within the electronic databases Medline, Embase, PsycINFO, Cinahl, and Web of Science in February 2020. After an initial search, the search strategy was amended to include relevant papers from personal libraries. The search was updated in March 2023. The search words were: ("compressed work*") OR (extend* shift*) OR (extend* adj (duty or work*) adj hour*) OR ((12* OR 10*) adj hour* adj2 "shift*"). See supplementary materials (appendix A) for the search strategy adapted to each database. Additional papers were included after screening the reference lists of included papers and relevant reviews (Banakhar 2017; Jill Clendon and Veronique Gibbons 2015; Estabrooks et al. 2009; Geiger-Brown and Trinkoff 2010; Harris et al. 2015; Kupperschmidt 2018; Sallinen and Kecklund 2010). Figure 1 shows the study selection.

Each title and abstract were independently reviewed by two researchers based on the selection criteria (see under) using the software Covidence.org ("Covidence systematic review software.,"). Relevant full-texts were retrieved and independently read in full text by two researchers. Cases of disagreement were discussed between the authors.

This systematic review was registered with International Prospective Register of Systematic Reviews (PROSPERO—CRD42020172595) (Bernstrøm et al. 2020). Guidelines of the Preferred Reporting Items for Systematic Reviews and Meta-Analyses Protocols (PRISMA-P) checklist (Larissa Shamseer 2015), and the “PICO”-approach were used (Schardt et al. 2007).

### Inclusion/exclusion criteria

Studies were included when the following criteria were met:

1. Participants: Employees whose weekly work hours did not exceed 55 h a week on average across the population. No limitations were put on work sector or employee group.
2. Intervention: Compressed work schedules.
3. Control: employees with "regular" workweeks (fixed day) or other shift arrangements, or pre-data.
4. Outcomes: Employee health/wellbeing (e.g., physical and mental health, stress, life satisfaction, sickness absence, work-life balance), and/or work outcomes including safety and performance outcomes (e.g. accidents, error/near error, adverse events, customer/patient satisfaction, and patients´ bed soars).
5. Method: Longitudinal quantitative studies (including both intervention and observational studies).
6. Journal, language and publication year: Full-text articles in peer-reviewed journals, published in English, Dutch or any Scandinavian language from 2005 to 2023.

For the compressed work schedule-criteria, we specifically included studies where (1) the authors clearly stated that they investigated a compressed schedule; (2) it was assessed as highly plausible from the description of the extended shifts that they were part of a compressed schedule (e.g. when a hospital ward changes from an 8-h shift system, to a 12-h shift system) or (3) outcomes of extended shifts were examined while controlling for weekly work hours. Studies were thus excluded when it was unclear whether they were part of a compressed schedule, and weekly work hours were not adjusted for. Studies focusing on a single shift (e.g. comparing fatigue or accidents after a specific shift) were therefore excluded as these studies did not account for the added days off.

To minimize erroneous rejection of studies, and get a better overview of the field, cross-sectional studies and studies investigating the consequences of extended shifts without controlling for weekly work hours were excluded during full-text screening and not abstract screening.

### Data extraction and quality assessment

Two authors independently extracted data into a standardized spreadsheet (including authors, publication year, sample, measures of work schedule and outcome variables, study design, and findings). Meta-analysis was not conducted due to heterogeneity of outcomes and study design.

The Effective Public Health Practice Project Quality Assessment (EPHPP) checklist (Armijo-Olivo et al. 2012) was used to assess the quality of the studies included. Two of the authors independently assessed the quality of each quantitative study. Studies were rated as strong, moderate or poor quality for six different components: (A) selection bias, (B) study design, (C) confounders, (D) blinding, (E) data collection methods and (F) withdrawals and drop-outs. Conflicts during extraction and quality assessment were discussed among screeners and raters until agreement was reached. Each study was then given a global score; strong (no weak component ratings), moderate (no more than one weak component rating), or weak (more than one weak rating). Furthermore, a total numeric score was given by transforming each component rating into scores: Strong = 3; Moderate = 2, Weak = 1, or N/A (not applicable) = 0, and summing component ratings, yielding a total score ranging from 5 to 18 for each study. The extracted data and quality assessments were used to discuss the risk of bias across studies.

### Evidence synthesis

Finally, we synthesized findings for each specific outcome following each study’s EPHPP- global rating, and the systematic review evidence synthesis guide (Breslin et al. 2019; Irvin et al. 2010; Slavin 1995). The guide ranges the level of evidence from strong to insufficient. Table 1 show the criteria for each level.

**Table 1:** Level of evidence based on EPHPP- global rating

Evidence	Rule (if the collective evidence meets one of the following criteria):
Strong	3 strong studies agree If more than three studies: ¾ of the moderate and strong studies agree
Moderate	2 strong studies agreeing 2 moderate studies and 1 strong study agree If more than three studies; more than 2/3 of the moderate and strong studies agree
Limited evidence	1 strong study 2 moderate studies 1 strong and 1 moderate agreeing If more than two studies, more than ½ of the moderate and strong studies agree
Mixed evidence	Moderate and strong studies showing contradictory results
Insufficient evidence	No strong studies, only 1 moderate study, and/or any number of weak studies

## Results

The database search resulted in 1580 references, as presented in Fig. 1. After excluding duplicates, 859 abstracts were screened by two independent researchers, of which 176 studies were also screened in full text. One additional study was included from reference lists in the included studies. Finally, 20 studies (see Table 2) met the inclusion criteria.

**Table 2:** Summary of studies included in the systematic analysis

Study	Sample (*N*, country); Data type	Study design; follow-up time	Exposure(s)	Outcome(s): health	Outcome(s): Safety and performance
Amendola et al. (2011)	Police officers (231, USA); Department records, simulator/test scores, and self-report	Randomized controlled trial; 6 months	CW: 8 vs 10 and 12	Sick leave, cardiovascular health, gastrointestinal problems, work stress, quality of personal life (work-family conflict), quality of work life (job satisfaction, schedule satisfaction, organizational commitment, and job involvement), sleep amount, sleep quality, sleepiness, alertness, and sleep disorder	Self-initiated activity (e.g. arrests), driving simulator, shooting simulator, fatigue/vigilance test (FIT and PVT), Interpersonal skill (B-PAD)
Bacon et al. (2005)	Industrial workers (*N* = 2802, UK); Self-reported	Intervention (two groups pre and post); 3 years	CW: 8h vs 12	Satisfaction with overall work hours, rota pattern, and overall job satisfaction Work pressure (high speed, deadlines, working under pressure, enough time, physically tired, mentally tired)	
Barnum (2011)	Police officers (NR^1^, USA); Department records	Simulation, and intervention (one group pre + post); NR	CW: 8 vs 10 vs 12	–	Time spent on calls
Battle and Temblett (2018a)	Nurses (*N* = 150, Wales); Department records, and self-report	Intervention (one group pre + post); 2 years (1 year after intervention)	CW: 8h (t1) vs 12h (t2)	Emotional exhaustion, depersonalization and personal accomplishment (MBI), sickness absence rate, personal injuries	Clinical incidents
Bell et al. (2015)	Police officers (*N* = 343, USA); Department records, simulator/test scores, saliva and self-report	Intervention (two groups pre + post); 9 months (1 to 6 months after intervention)	CW: 13:20hx3days vs 10hx4days	Sleep and daytime dysfunction (PSQI), stress (Salivary cortisol), Quality of life (QOLI), partner QoL (partner-report), sickness absence (department records)	Attention/vigilance (PVT), cognitive performance (STROOP test), Pass/fail shooting qualification, citizen complaints, officer involved accidents, and activity data (self-initiated calls, bookings, field interrogations) (department records)
Casjens et al. (2022a)	Industrial workers (*N* = 129, Germany); Self-report and Actigraphy	Longitudinal; 4 weeks	CW: 8h vs 12 (comparing two 8-h shift patterns, two 12-h shift pattern, and one permanent night shift)	Sleep duration Sleep debt (the absolute difference of sleep duration between workdays and work-free days) Social jetlag (the difference between mid-sleep on workdays and work-free days – a proxy for circadian misalignment. Mid sleep is the midpoint of the main sleep episode) Sleep quality (Locomotor Inactivity During Sleep)	
Casjens et al. (2022b)	Industrial workers (*n* = 95, Germany); Hair cortisol concentration	Longitudinal; 3 years (yearly measure)	CW: 8h vs 12	Stress (Hair cortisol)	
Dall'Ora et al. (2019)	Nurses and health care assistants (*N* = 1944, UK); Department records	Longitudinal; Data collected over 3 years with 7 day exposure- windows	Extended shifts (≥ 12 h) controlled for days worked	Sickness absence (total, short 1–6 days, and long > 6 days)	-
Dionne and Dostie (2007)	Employees (*N* = 18 671, Canada); Self-report (WES)	Longitudinal; 3 years (analyses include yearly measures of CW and absence)	CW (self-reported, hours not specified)	Total number of days absent during the year (i.e. all paid and unpaid leave)	-
Mills and Grotto (2017)	Senior executives at a technology company (*N* = 133, USA); Self-report and supervisor ratings	Longitudinal; 4 months (T2) and 9 months (T3)	CW (self-reported, hours not specified)	–	Supervisor-rated performance (T2) and self-reported organizational commitment (T3)
Oh and Yim (2018)	Industrial workers (*N* = 2090, South-Korea); physician rated	Longitudinal; 5 years (T1 2010, and T2 2015)	CW: daytime workers, three-shift with night (8 h), and compressed two- shift with night (12 h) system	Metabolic syndrome	–
Ooi et al. (2021)	Radiographers’ (*N* = 48 employees and 15,0277 images acquired, Singapore); Department records	Intervention (one group pre and post); 1 year post intervention was during Covid	CW: 2 shift system (12h) vs 3 shift system (6h day + 7h evening + 11 h night)		Image rejection
Puttonen et al. (2022)	Industrial workers (n = 178, Finland); Self-reported	Intervention (two groups pre and post); follow-up 9–12 months after intervention	CW: 8h vs 12h shift system	Sleep length (habitual sleep length, sleep length by shift), Sleepiness (morning and night shift), Insomnia (morning and night shift), subjective health, work ability, need for recovery, satisfaction with shift system, perceived negative effect of shift system (on sleep and alertness, work-life balance, fluency at work, commuting)	
Rodriguez Santana et al. (2020)	Nurses and health care assistants (N = 6 wards, England); Department records	Intervention (6 groups pre and post); groups implemented at 3 different times: follow-up 5 to 8 months after intervention	CW: 8h vs 12h shift system	Short-term sickness absence (< = 7 days)	
Ropponen et al. (2020)	Hospital employees (*N* = 21,440, Finland); Department records	Longitudinal (case-crossover design); 10 years: 28 day exposure-window prior to sickness absence incident and control window (no absence)	Extended shifts (≥ 12 h) while controlling for weekly and daily work hours	Short-term sickness absence	–
Shochat et al. (2019)	Airline ground crew managers (*N* = 39, Israel); Self- reported and Actigraphy	Intervention (one group pre and post) ; follow up 3 months after intervention	CW: 8h vs 12h shift system;	Burnout (SMBM), Sleep Quality (PSQI), Sleep length and efficiency for day sleep, nights sleep and naps (actigraphy), Sleepiness (KSS), Caffeine intake	
Su et al. (2008)	Female high-tech employees (*N* = 229, Taiwan); Self-report	Longitudinal; 9 months (with monthly measures)	CW: rotating 12 h shift- workers with night shifts compared to regular day workers	Menstrual cycle irregularities	–
Tanaka et al. (2010)	Nurses (*N* = 1 407, Japan); Self-report	Longitudinal; 6 months	CW: 2 shift system (9 h day + 16 h night) vs 3 shift system (8.5h day + 8.5h evening + 10 h night)	-	Adverse events (defined as incidents in which the subject made an error that resulted in harm to patient during the last 6 months)
Trinkoff et al. (2006)	Nurses (*N* = 2617, USA); Self-reported	Longitudinal; 15 months (T2 at 6 months, and T3 at 15 months)	Extended shifts (≥ 13 h) while controlling for daily, and weekly work hours and days worked a week	Musculoskeletal disorder (neck, shoulder, back-pain symptoms)	–
Wijaya et al. (2020)	Hospital employees (*N* = 356, Indonesia); Self-reported	Intervention (two groups pre and post); 9 months (8 months after intervention)	CW: approx. 8h vs 12h (the 12h intervention also including constant shift starting time across hospital departments)	–	Patient safety culture

Sample refers to number of participants according to response rate. Only exposures and outcomes tested for statistical significance are presented in Table 2. ^1^NR = not relevant due to simulation study

*CW* compressed work schedule

All studies were published in English between 2005 and 2022. Sample sizes ranged from 39 to 21 440. Most of the 20 studies examined health care workers (n = 8) or industrial workers (n = 5), with the rest examining workers within the police (n = 3), technology (n = 2) and airport (n = 1). One study examined compressed workweeks in the general working population. The studies were from Europe (n = 8), North America (n = 6), Asia (n = 5) and The Middle East (n = 1). The study design was longitudinal designs with follow-up intervals varying from 4 weeks to 10 years.

**Fig. 2:**
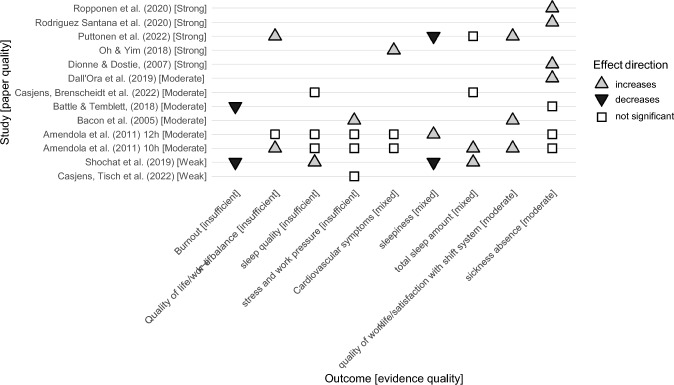
Evidence map health

**Fig. 3:**
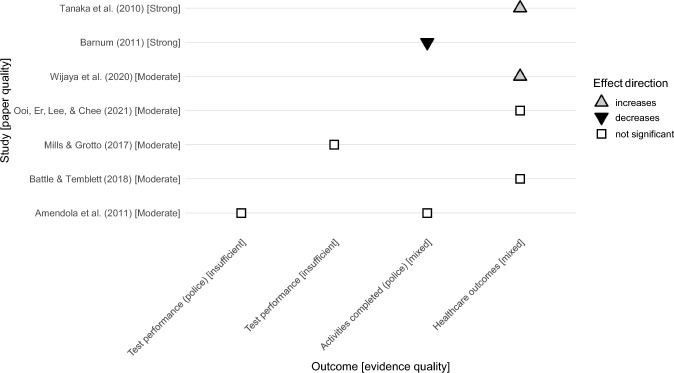
Evidence map job outcomes

Of 20 studies, 15 studies investigated outcomes related to employee health and wellbeing (Table 3), and 8 studies related to work safety and performance (Table 4). See Table 5 for the results of the quality assessment of the included studies. Below we synthesise the evidence based on the type of outcome, shift length and work hours in the control group, and summarize the quality of evidence. The evidence synthesis based on outcome is illustrated in Fig. 2 (health and wellbeing) and Fig. 3 (safety and performance).

**Table 3:** Health outcomes related to compressed work schedules

Compressed work versus normal work	Findings	Source
CWW: 10 h versus 8 h	CW better than nCW: Quality of work life CW better than nCW: Sleep amount No significant difference: Sick leave, cardiovascular health, gastrointestinal problems, work stress, quality of personal life, sleep quality, sleep disorder Not reported if significant different: sleepiness and alertness	Amendola et al. (2011)
CWW: 12 h versus 8 h	CW worse than nCW: Sleepiness and alertness No significant difference: Sick leave, cardiovascular health, gastrointestinal problems, work stress, quality of personal life, sleep quality, sleep disorder Not reported if significant different: Quality of work life and sleep amount	
CWW: 12 h versus 8 h	CWW better than nCW: satisfaction with working hours, satisfaction with rota pattern and overall job satisfaction CW worse than nCW: Work pressure (high speed, deadlines, working under pressure, and physically tired) No significant difference: Work pressure (enough time, and mentally tired)	Bacon et al. (2005)
CWW: 12 h versus 8 h	CWW better than nCW: emotional exhaustion and depersonalization No significant difference: personal accomplishment, sickness absence rate, personal injuries	Battle and Temblett (2018a))
CWW: 13,12 h × 3 days versus 10 h × 4 days	3 day CWW worse than 4 day CWW: sleep (quantity and quality), daytime dysfunction, quality of life No significant difference: sickness absence, stress, partner quality of life	Bell et al. (2015)
CWW: 12 h versus 8 h	CW better than nCW: Sleep duration on work-free days CW worse than nCW: Sleep duration on workdays, social jetlag, sleep debt No significant difference: Overall sleep duration, sleep quality	Casjens et al. (2022a)
CWW: 12 h versus 8 h	No significant difference: median hair cortisol concentration or change in hair cortisol concentration during covid-19	Casjens Tisch et al. (2022b)
Extended shifts ≥ 12 h versus ≤ 8 h	Extended shifts worse than 8 h: sickness absence (both long- and short-term)	Dall'Ora et al. (2019)
CWW versus 8 h (CWW hours not specified)	CWW worse than nCW: number of absence days	Dionne and Dostie (2007)
CWW: 12 h (two- shift with night) versus 8 h (three-shift with night) and 8 h day-shift	CWW worse than nCW: metabolic syndrome	Oh and Yim (2018)
CWW: 12 h versus 8 h	CW better than nCW: Sleep length before and between morning shifts, sleepiness in morning shifts and night shifts (measure 1), satisfaction with shift system, perceived negative effects of shifts system on sleep and alertness, and work life balance No significant difference: Habitual sleep length, sleepiness in morning shifts and night shifts (measure 2), Sleep length before night shift, after night shift, after last night shift, between days off, Insomnia in relation to morning and night shifts, subjective health, work ability, need for recovery, perceived negative effects of shifts system on fluency of work and commuting	Puttonen et al. (2022)
Extended shifts ≥ 12 h	Extended shifts worse: short-term sickness absence for young employees (ages ≤ 25 years) No significant difference: short-term sickness absence for employers > 25years	Ropponen et al. (2020)
CWW: 12 h versus 8 h	CW worse than nCW: short-term sickness absence	Rodriguez Santana et al. (2020)
CWW: 12 h versus 8 h	CW better than nCW: Burnout (physical strength, vitality thinking, mental energy), subjective sleep quality, sleep time and efficiency during naps measured by actigraphy, sleepiness during day shift, CW worse than nCW: sleepiness during night shift at 03:00 No significant difference: sleep time and efficiency during main sleep episode measured by actigraphy, sleepiness during night shift at a 00:00 – 02:00 and 04:00– 07:00	Shochat et al. (2019)
CWW: Rotating 12 h with night shift compared to regular day	CWW worse than nCW: menstrual cycle irregularity	Su et al. (2008)
Extended shift ≥ 13 h	Extended shifts worse: musculoskeletal disorder	Trinkoff et al. (2006)

*CW compressed work schedule, *nCW* non-compressed work schedule. Only exposures and outcomes fulfilling inclusion criteria, and tested for statistical significance are presented

**Table 4:** Safety and performance outcomes related to compressed work schedules

Compressed work versus normal work	Findings	Source
CWW: 10 h versus 12 h versus 8 h	No significant difference: self-initiated activity, driving and shooting simulators, fatigue/vigilance test and interpersonal skill	Amendola et al. (2011)
CWW: 12 h versus 8 h	CWW worse than nCWW (12 h): time held on calls for service No significant difference: time held handling calls once they were dispatched	Barnum (2011)
CWW: 12 h versus 10 h vs 8 h	CWW worse than nCWW: staffing (match between fluctuation in call volume and percentage of officers working at any given hour)	
CWW: 12 h versus 8 h	No significant difference: clinical incidents	Battle and Temblett (2018a)
CWW: 13,12 h × 3 days versus 10 h × 4 days	3 day CWW worse than 4 day CWW: reaction time, concentration, cognitive processing, citizen complaints, officers involved in accidents 3 day CWW better than 4 day CWW: anticipatory error, more adult bookings and field interrogations No significant difference: shooting qualification, self-initiated calls	Bell et al. (2015)
CWW (self-reported, hours not specified)	No significant difference: performance, organizational commitment	Mills and Grotto (2017)
CWW: 2 shift system (12h) vs 3 shift system (6h day + 7h evening + 11 h night)	CWW better than nCWW: X-ray image reject count No significant difference: X-ray image reject rate and total count	Ooi et al. (2021)
CWW: 2 shift system (9 h day + 16 h night) vs 3 shift system (8.5h day + 8.5h evening + 10 h night)	CWW in 2 shift system better than nCWW in 3 shift system: adverse events	Tanaka et al. (2010)
CWW: 12 h versus 8 h (the 12 h-intervention also included constant shift starting time across hospital departments)	CWW (with aligned shift schedule across departments) better than nCWW: patient safety culture	Wijaya et al. (2020)

*Number 1, 2 and 3 in the first column refers to studies primarily focusing on health (1), safety (2) and performance (3). *CWS* compressed work schedule., *nCWS* non-compressed work schedule

**Table 5:** Quality assessment of studies based on EPHPP criteria: Total score and quality according to component ratings

Source, Country	Total score (5–18)	Global rating	Selection bias	Study design	Control of confounders	Blinding	Data collection methods	Withdrawals and drop-outs
Amendola et al. (2011)	13	Moderate	Weak	Strong	Strong	Week	Strong	Strong
Bacon et al. (2005)	9	Moderate	Moderate	Moderate	Moderate	N/A	Moderate	Weak
Barnum (2011)	14	Strong	Moderate	Moderate	Moderate	Strong	Strong	Moderate
Battle and Temblett (2018a)	10	Moderate	Moderate	Moderate	Moderate	N/A	Strong	Moderate
Bell et al. (2015)	13	Strong	Moderate	Strong	Strong	Weak	Strong	Moderate
Casjens et al. (2022a)	11	Moderate	Weak	Moderate	Moderate	N/A	Strong	Strong
Casjens et al. (2022b)	8	Weak	Weak	Moderate	Weak	N	Strong	Weak
Dall'Ora et al. (2019)	10	Moderate	Moderate	Moderate	Weak	N/A	Strong	Moderate
Dionne and Dostie (2007)	13	Strong	Moderate	Moderate	Strong	N/A	Strong	Strong
Mills and Grotto (2017)	11	Moderate	Moderate	Moderate	Strong	N/A	Strong	Weak
Oh and Yim (2018)	12	Strong	Moderate	Moderate	Strong	N/A	Strong	Moderate
Ooi et al. (2021)	11	Moderate	Moderate	Moderate	Weak	N/A	Strong	Strong
Puttonen et al. (2022)	11	Strong	Moderate	Moderate	Strong	N/A	Moderate	Moderate
Rodriguez Santana et al. (2020)	13	Strong	Moderate	Moderate	Strong	N/A	Strong	Strong
Ropponen et al. (2020)	13	Strong	Moderate	Moderate	Strong	N/A	Strong	Moderate
Shochat et al. (2019)	9	Weak	Weak	Moderate	Moderate	N/A	Strong	Weak
Su et al. (2008)	12	Strong	Moderate	Moderate	Strong	N/A	Strong	Moderate
Tanaka et al. (2010)	15	Strong	Moderate	Moderate	Strong	N/A	Strong	Strong
Trinkoff et al. (2006)	13	Strong	Moderate	Moderate	Strong	N/A	Strong	Strong
Wijaya et al. (2020)	12	Moderate	Moderate	Strong	Moderate	Weak	Strong	Weak

^*^Blinding-criteria did not count in terms of global score because it is impossible to blind the study’s exposure variable (how many hours one works a week). Total score computed by converting strong = 3, moderate = 2, weak = 1, and N/A = 0, and computing a sum score. Global rating was assigned according to the EPHPP guidelines (strong = no weak ratings, moderate = one weak rating, weak = two or more weak ratings). Withdrawals and drop-outs was assessed as moderate (and not weak) in studies who did not adequately report drop out after EPHPP guidelines when the study used department records or equivalent registry data. Department records include all employees at the workplace and withdrawals can be assumed to be limited to employees leaving their current employer/workplace

### Health and wellbeing outcomes

Among the 15 studies investigating health and wellbeing outcomes, 11 studies compared a compressed schedule to an 8 h schedule, 3 studies investigated extended shifts while controlling for weekly work hours, and one study compared two different compressed schedules (3 days of 13.5 vs 4 days of 10 days). The most frequently investigated health outcomes were sickness absence and sleep, with a total of 6 studies investigating each outcome. The results are illustrated in Fig. 2 (sorted by quality of evidence and including only outcomes investigated in at least two studies).

***In sum,*** the results were mixed. Of 15 studies, 11 reported at least one significantly negative health or wellbeing outcome of a compressed schedule or extended shifts, 10 studies reported at least one non-significant outcome, and 6 studies reported at least one significantly positive outcome. Of the outcomes examined in multiple studies we found moderate support for compressed schedules being beneficial for satisfaction with work schedule. We found moderate support for compressed schedule (and particularly extended shifts (≥ 12 h) controlled for weekly work hours) being detrimental in terms of sickness absence. For all other health and wellbeing outcomes the results were either mixed or insufficient for conclusions.

Reviewing the specific outcomes in more detail; of the seven studies investigating ***sickness absence***, two found no significant difference, four found higher absence among the compressed workers and one compared two different compressed schedules. The two studies which found no significant differences both compared a compressed schedule with 12 h shift to a non-compressed schedule (Amendola et al. 2011; Battle and Temblett 2018b), one of the studies also included a 10 h shift schedule (Amendola et al. 2011). Of the four studies that found higher absence rates, one compared a compressed 12 h shift schedule to an 8 h shift schedule (Rodriguez Santana et al. 2020), two investigated extended shifts (≥ 12 h) controlled for weekly work hours (Dall'Ora et al. 2019; Ropponen, Koskinen, Puttonen, & Harma, 2020), and one investigated self-reported compressed work schedules without specifying length of shift (Dionne and Dostie 2007). The last study compared two different compressed schedules (13:20 h × 3 days vs 10 h × 4 days) and found no significant differences (Bell et al. 2015). Because Bell et al. (2015) compares two compressed schedules this study is not included in the evidence synthesizing using the EPHPP-global rating. Overall, there is moderate support that compressed work schedules are longitudinally linked with higher sickness absence, according to the EPHPP-global rating.

Of the six studies investigating ***sleep***, all have included different sleep related outcomes, most commonly sleep quality, sleep amount, and sleepiness.

One study found partial support for better *sleep quality* (Shochat et al. 2019), while two studies found no significant difference in sleep quality (Amendola et al. 2011; Casjens et al. 2022a), all examining compressed 12 h shift schedule versus non-compressed shifts. Moreover, one study also found no significant difference in sleep quality when examining a compressed 10 h shift schedule versus non-compressed shifts (Amendola et al. 2011). A fourth study compared two different compressed schedules and found poorer sleep quality when employees work 13.5 h shifts rather than 10 h shifts (Bell et al. 2015). There is insufficient evidence to support a longitudinal relationship between compressed work and sleep quality, according to the EPHPP-global rating.

Investigating *sleep amount*, two studies found that employees reported more sleep in total when working a compressed 12 h (Shochat et al. 2019) and 10 h schedule (Amendola et al. 2011) versus a non-compressed schedule. The difference was not significant when sleep was measured objectively by actigraphy in one of these studies (Shochat et al. 2019). Two studies found no significant difference in total or habitual sleep, but they found significant differences when dividing sleep according to shift type and work-free days (Casjens et al. 2022b; Puttonen et al. 2022). More specifically, one study found that employees working a compressed schedule with 12 h shift, compared to a non-compressed schedule, slept more on work-free days, but less on workdays, yielding insignificant difference on total sleep (Casjens et al. 2022a). The other study found that employees on compressed schedules of 12 h shift slept more before and between morning shifts compared to employees on a non-compressed schedule, but not on any other day (Puttonen et al. 2022). Finally, one study found more sleep when employees worked a compressed schedule of 10 h shifts compared to a compressed schedule of 13.5 h shifts (Bell et al. 2015). There is mixed evidence for a relationship between compressed work and sleep amount.

Four studies investigated *sleepiness, alertness and daytime disfunction*. Two studies found partial support for less sleepiness (Puttonen et al. 2022; Shochat et al. 2019) while another found significantly more sleepiness and less alertness (Amendola et al. 2011) when working a compressed 12 h schedule, as compared to a non-compressed schedule. Comparing two compressed schedules, a fourth study found more daytime dysfunction due to sleepiness (e.g. having trouble staying awake) when employees work 13.5 h shifts rather than 10 h shifts (Bell et al. 2015). There is mixed evidence for a relationship between compressed work and sleepiness.

Of additional sleep related outcomes two studies found no significant difference in sleep disorder between a compressed 12 h schedule, 10 h schedule and non-compressed schedule (Amendola et al. 2011) or insomnia for a compressed 12 h schedule compared to a non-compressed schedule (Puttonen et al. 2022). One study found that employees working compressed 12 h shifts had significantly more social jetlag (i.e. a larger difference in sleep patterns between work and free days) (Casjens et al. 2022b).

Four studies investigated ***stress and work pressure***. Of these, two studies found no significant difference in stress measured with cortisol levels between 12 and 8 h shift workers, (Casjens et al. 2022a) or between 13 and 10 h shift workers (Bell et al. 2015). One study found no significant difference in work-related stress measured with self-reports between 12 h, 10 h and 8 h shift workers (Amendola et al. 2011). One study found significant higher self-reported work pressure for employees working a compressed schedule of 12 h (Bacon et al. 2005). There is insufficient evidence to support a relationship between compressed work and stress and work pressure. Rather, with three strong or moderate studies reporting no difference in stress or work pressure, the evidence could be interpreted as moderate support for there not being a relationship. However, it is important to note that none of the studies used non-inferiority tests or equivalent approaches to directly assess the certainty of the null hypothesis (i.e. that there is no difference).

Three studies investigated ***quality of work life, job satisfaction and satisfaction with shift system***. All studies reported positive results for a compressed schedule. The results included higher satisfaction with this shift system (Puttonen et al. 2022) and a higher satisfaction with working hours, shift rotation pattern and overall job satisfaction (Bacon et al. 2005) among employees working a compressed 12 h shift schedule compared to a non-compressed schedule. In addition, employees working a compressed 10 h shift schedule reported better quality of work life (measured as job satisfaction, schedule satisfaction, organizational commitment, and job involvement) compared to a non-compressed schedule (Amendola et al. 2011). In accordance with the EPHPP-global rating, there is moderate evidence that compressed work schedules are longitudinally linked with higher satisfaction with shift system.

Three studies investigated ***quality of life or work-life balance***. One study found significantly improved work-life balance among employees working a compressed 12 h schedule (Puttonen et al. 2022). Another study reported no significant differences in self-reported quality in personal life (measured as work-family conflict) when working a compressed 12 or 10 h schedule compared to a non-compressed schedule (Amendola et al. 2011). A final study reported significantly lower quality of life among employees working a compressed schedule with 13.25-h shifts compared to employees working 10 h shifts, and no significant difference in partners’ quality of life (Bell et al. 2015). The evidence for work-life balance is insufficient.

Two studies investigated ***burnout***—both supporting less burnout among employees working compressed 12 h work schedules (Battle and Temblett 2018b; Shochat et al. 2019). With one study rated as weak and one moderate—the evidence for burnout is insufficient.

Two studies investigated ***cardiovascular health***, with one study finding no significant difference in cardiovascular health between compressed 12 h, 10 h and a non-compressed schedule (Amendola et al. 2011) and the other finding increased risk of metabolic syndrome for employees working compressed 12 h work schedules (Oh and Yim 2018). The evidence for cardiovascular health is therefore mixed.

Finally, certain outcomes were only investigated in one study supporting negative consequences for health outcomes including menstrual cycle irregularities (Su et al. 2008) and musculoskeletal disorder (Trinkoff et al. 2006), while others reported no significant differences for gastrointestinal problems (Amendola et al. 2011), personal injuries (Battle and Temblett 2018b), subjective health, work ability, and need for recovery (Puttonen et al. 2022).

### Safety and performance outcomes

In all, eight studies investigated safety or performance outcomes of compressed work weeks. The outcome measures were diverse. Several studies focus on sector-specific outcomes, particularly outcomes in the healthcare sector and police force. The results are illustrated in Fig. 2 (sorted by quality of evidence).

***In sum,*** the studies revealed mixed results for the safety and performance consequences of compressed workweeks when outcomes are grouped together. The level of evidence for each individual outcome is insufficient—with few studies investigating the same outcome. Two studies reported significantly positive outcomes (Tanaka et al. 2010; Wijaya et al. 2020), and one studies reported significantly negative outcomes (Barnum 2011). And one study reported mixed results when comparing compressed shifts of different lengths (Bell et al. 2015). Most studies found some insignificant outcome measures.

Reviewing the specific outcomes in more detail; four studies investigated safety and performance outcomes of compressed workweeks within the ***healthcare setting***. Two studies found beneficial outcomes for patient safety; including improved patient safety culture when comparing a compressed schedule of 12 h shifts to 8 h shifts (Wijaya et al. 2020) and reduced risk of error in patient care when comparing a compressed schedule of 9 h day and 16 h night to a schedule of 8 h day and 10 h night (Tanaka et al. 2010). Two other studies found no significant differences in clinical incidences (i.e. events that could have, or did result in, unnecessary harm) (Battle and Temblett 2018b) and the rate of x-ray images taken by radiographers that had to be rejected (Ooi et al. 2021) when comparing a compressed schedule with 12 h shifts to a non-compressed shift schedule. Both studies supporting positive effects of a compressed workweek on patient safety relied solely on self-report. There is mixed evidence for whether a compressed work schedules is longitudinally linked with healthcare outcomes. Though mixed, findings are positive or non-significant, and there are no studies supporting negative health care outcomes of compressed workweeks.

Three studies investigated safety or performance outcomes of compressed workweeks in the ***police force***. All three studies included some measure of *activities completed.* One study found no significant difference in activity levels (e.g. arrests made) between 12, 10 and 8 h shifts (Amendola et al. 2011). The second study found significant longer time for answering calls during 12 h shifts compared to 8 h shifts, but no other significant difference in time spent handling calls (Barnum 2011). The third study found that police officers working 13 h shifts conducted significantly more adult bookings (i.e., the processing of individuals taken into custody) and field interrogations compared to those working 10 h shifts (Bell et al. 2015). There is mixed evidence for whether a compressed work schedule is longitudinally linked with activities completed withing the police force.

Two of the studies investigating safety or performance in the police force also included *performance on test and simulations, one comparing a comparing two compressed schedule of 12 h and 10 h shifts to an* 8 h hour shift schedule (Amendola et al. 2011) and one comparing between 13 and 10 h schedules (Bell et al. 2015). The studies did not find a significant difference in police officers´ results on shooting tests between any of the schedules (Amendola et al. 2011; Bell et al. 2015) or on a driving simulator (Amendola et al. 2011). One study found no significant difference on vigilance or fatigue test (Amendola et al. 2011), while another study showed mixed results of the vigilance test as officers working 13 h shifts demonstrated significantly more lapses in concentration compared to officers on 10 h shifts, but also fewer anticipatory errors at one point after the intervention (Bell et al. 2015). The latter study examining vigilance also found no significant difference between 13 and 10 h shift schedules in cognitive processing at the beginning of a shift, but significantly poorer cognitive processing at the end of shift among officers working the longest shifts (Bell et al. 2015). Additionally, they found that officers working the 13 h shift schedule had a significant increase in number of citizen complaints, and involvement in incidents (Bell et al. 2015). There is not sufficient evidence to assess whether a compressed work schedule is longitudinally linked with performance on test and simulation.

Finally, as the only study investigating safety and performance outcomes outside the health and police sectors; investigating senior executives in a technology company, Mills and Grotto (2017) found no significant relationship between self-report of working compressed workweeks and supervisor-rated performance.

### Summarizing all findings by shift length and work hours in control groups

Here we summarize all findings about the consequences of compressed workweeks by the shift length and alternative schedules. The compressed workweeks were generally operationalized as shift lengths of 12 or 10 h, though some had alternative schedules or unspecified length. The studies were conducted in different professional settings, but the majority, were conducted on shift workers (n = 16 of 20 studies). In total, seven studies focused on healthcare workers, five focused on industrial workers, and four investigated employees in the police force. In these studies, both the compressed workweek employees and the control group worked shift schedules making up more than 8 h staffing (e.g. a 12 h day shift compared to an 8 h rotating day and evening shift).

In sum, the evidence for a compressed schedule with 12 h shifts compared to an 8 h shiftwork schedule were mixed, with studies supporting both positive and negative health and performance outcomes. In comparison, the results for ≥ 12 h shifts controlling for weekly work hours all supported negative health consequences. Only two studies investigated a compressed schedule with 10 h shifts, however they cautiously indicated positive health and performance consequences of a compressed schedule with 10 h shifts compared to a compressed schedule with 12 h shifts or a traditional 8 h shift schedule. Finally, the evidence for a compressed schedule compared to fixed day supported negative health consequences.

Reviewing the shifts lengths in more detail; eight studies investigated health outcomes of a compressed *12 h-schedule compared to 8 h shift schedules*. Of these studies, three showed positive health and wellbeing outcomes of compressed 12 h work schedules (Battle and Temblett 2018b; Puttonen et al. 2022; Shochat et al. 2019), two supported poorer health and wellbeing (Amendola et al. 2011; Rodriguez Santana et al. 2020), two showed mixed results (Bacon et al. 2005; Casjens et al. 2022a), and one found no significant difference (Casjens et al. 2022b). Four studies investigated safety and performance outcomes of a compressed 12 h-schedule compared to 8 h shift schedules. Of these, one showed a positive outcome (Wijaya et al. 2020), one a negative (Barnum 2011), and two showed primarily no significant difference (Amendola et al. 2011; Battle and Temblett 2018b). Collectively, the results of a compressed 12 h schedule compared to 8 h shift schedules on both health and performance consequences are mixed.

Three studies *investigated frequencies of* ≥ *12 or* ≥ *13 h shift*, not explicitly examining compressed workweek schedules, while controlling for weekly work hours, all with negative health outcomes (Dall'Ora et al. 2019; Ropponen et al. 2020; Trinkoff et al. 2006).

Two studies investigated *10 h shift schedules compared to other schedules*. One study reported improved health indicators among employees working 10 h shift compared to 8 and 12 h shifts (Amendola et al. 2011). The second study found both health and performance advantage of 10 h shift compared to longer shifts, but made no comparison to traditional 8 h shifts (Bell et al. 2015). Results for 10 h shift are thus more positive than for 12 h shift, however, there is a clear lack of studies comparing 10 h shifts to a traditional 8 h shift schedule.

Four studies investigated professional settings in which a *control group mainly included traditional day workers*. The studies include female tech employees (Su et al. 2008), senior executives at a technology company (Mills and Grotto 2017), manufacturing employees (Oh and Yim 2018), and employees in general (Dionne and Dostie 2007). For these studies the compressed schedule included either 12 h shifts (Oh and Yim 2018; Su et al. 2008), or self-reported and unspecified compressed schedules (Dionne and Dostie 2007; Mills and Grotto 2017). Three of the studies reported adverse health consequences of working compressed schedules compared to fixed day (Dionne and Dostie 2007; Oh and Yim 2018; Su et al. 2008) and one study found no significant difference of performance and organizational outcome (Mills and Grotto 2017). In general, there is limited evidence on the potential beneficial or adverse consequences of working compressed work schedules in sectors not requiring shift work. However, the few existing studies are predominantly pointing towards adverse health consequences.

### Quality and methodological approaches

The quality assessment of each study is presented in Table 5. Of the 20 included studies 10 (50%) were assessed as having a strong global rating, 8 (40%) a moderate global rating, and two (10%) a weak global rating. Here we discuss the risk of bias for each subcategory (selection bias, study design, data collection, withdrawals).

Risk of selection bias was rated as moderate in all but two studies (i.e. 90% of included studies), the latter two were categorized as weak. All but one study focused on sector specific populations, and several studies included only one or a few organizations—limiting the generalizability of the findings. However, six studies utilized existing records, such as department records. By using existing records (e.g., covering all employees in each department) the studies eliminate selection biases connected to those who choose to participate in the study.

In three studies (15% of included studies) the study design was categorized as strong (i.e. randomized control trial or controlled clinical trial). These studies found positive performance outcomes of 12 h shifts compared to 8 h shift (Wijaya et al. 2020), partially negative health and performance consequences of 12 h shifts compared to 8 h shifts (Amendola et al. 2011), and positive health and performance consequences of 10 h shifts compared to both 8 h shifts (Amendola et al. 2011) and 12/13 h shifts (Amendola et al. 2011; Bell et al. 2015).

There were seven additional intervention studies (35% of included studies), categorized as moderate in terms of study design quality as they did not meet the EPHPP’s criteria for a controlled trial. Of these, three studies found predominantly positive health outcomes from 12 h shift (Battle and Temblett 2018b; Puttonen et al. 2022; Shochat et al. 2019), one found negative health outcomes from 12 h shift (Rodriguez Santana et al. 2020), and one found mixed wellbeing outcomes from 12 h shift (Bacon et al. 2005). One study also found one negative performance outcome (Barnum 2011), while two found no significant difference in performance (Battle and Temblett 2018b; Ooi et al. 2021).

All but two studies were categorized as strong on data collection methods (90% of included studies), indicating that they used valid and reliable measures for both schedule and outcome measures. Noticeably, several studies used one-item self-report measures of work schedule. We coded these studies as having a valid measure of work schedule, despite being single items and not previously validated. Arguably, most employees have a good understanding of whether they are scheduled to work 8, 10 or 12 h shifts.

The degree of withdrawals and drop-outs varied between studies, and in several studies, it was difficult to assess or not applicable. Importantly, for several studies relying on existing records (e.g. department records), withdrawals from the study are equivalent to individuals leaving the study population (e.g. employees changing jobs).

Follow up time from measure of exposure varied from 7 days to 5 years, with several studies focusing on a follow up time between 6 months and 1 year.

## Discussion

In the current systematic literature review, we assessed the longitudinal employee and workplace consequences of compressed workweeks. We identified 20 longitudinal studies examining the consequences of compressed workweeks directly, or indirectly with extended daily work hours while controlling for weekly work hours. The results were mixed for both health and performance outcomes, and suggested that findings vary depending on the type of compressed schedule, shift length and type of work hour arrangement serving as comparison. The results yielded moderate support for satisfaction with shift system.

In their systematic literature review Bambra et al. (2008) concluded that compressed work can improve work-life balance, and that it may do so with a low risk of adverse health or organizational effects. The results of the current review, including only longitudinal studies were more mixed, and less optimistic.

Most of the studies in the current review focused on health and well-being outcomes. Unlike the review by Bambra et al. (2008), there were slightly more studies finding significant negative health and wellbeing outcomes than positive health outcomes of compressed work schedules. Specifically, the results were cautiously negative for sickness absence (i.e. more sickness absence for compressed workers), and cautiously positive for satisfaction with work schedule. In line with former reviews (Driscoll et al. 2007), our results were primarily mixed for sleep and sleepiness outcomes, though some indication of increased sleep amount for employees working a compressed week at least on some nights. In comparison to the current review, Bambra et al. (2008) focused solely on experimental and quasi-experimental studies. The current review was broader, including also longitudinal observational studies. However, limiting our findings to those from intervention studies still yields highly mixed results.

When looking at the differences in work schedules; studies comparing a compressed workweek to fixed day work supported negative health outcomes. Similarly, studies investigating frequency of 12 h shifts not specifically organized in a compressed schedule were also predominantly negative. In contrast, studies comparing a compressed workweek of 12 h shift to other shift arrangements were highly mixed, while studies comparing 10 h shift to other shift arrangements were few but more positive.

In sum, the results for health and well-being outcomes are highly mixed, and possibly dependent on the specific outcome, length of extended shifts, and work hour arrangement in the control group. It is also possible that employees working a compressed week experience some negative health consequences from working extended shifts, while also benefiting from extended periods of recovery. How the free periods are organized would then also be of importance.

Less than half the studies included performance and safety outcomes. They primarily focused on outcomes within a specific workplace sector, particularly the healthcare sector and police force. In general, the results for performance and safety were mixed, with studies supporting both positive and negative outcomes of a compressed workweek, as well as multiple studies identifying no significant difference. The outcomes investigated were highly heterogeneous though, which makes the mixed results difficult to aggregate and compare.

When looking at sector specific outcomes, the results for activities and performance in the police force were mixed, with both positive and negative outcomes. However, the studies on healthcare outcomes yielded either positive or insignificant differences for patient safety, and thus more in line with Bambra et al. (2008) indicating that compressed workweeks may be implemented without adverse organizational outcomes.

Prior research on extended shifts have supported that increased fatigue towards the end of a long shift may lead to adverse job outcomes such as an increased risk of errors (Bae & Fabry 2014; Clendon and Gibbons 2015; Dall'Ora et al. 2016; Leroyer et al. 2014). However, the relationship between a compressed workweek and performance outcomes may be more complex. Within the healthcare sector handovers, when the responsibility for a patient is transferred from one person to another, is considered an important risk of error (Desmedt et al. 2020). By extending the length of a shift the number of handovers necessary during 24 h is reduced. Within the police force simulations have demonstrated that the length of the shift is also important for efficient use of personnel (i.e. match between fluctuation work demand and percentage of officers working at any given hour) (Barnum 2011). It is therefore important to investigate the total effects of a compressed workweek on performance and safety outcomes. There is a clear need for more high-quality studies investigating the potentially complex relationship between compressed workweek and patient and safety outcomes.

In sum, while the results of the current review on compressed workweek were not as optimistic as those of Bambra et al. (2008), the results are not as negative as prior reviews investigating extended shifts (Bae and Fabry 2014; Banakhar 2017; Bannai and Tamakoshi 2014a, b; Dall'Ora et al. 2016; Kang et al. 2012; Solovieva et al. 2013). Our results indicate that the consequences of compressed workweeks are complex, and that an important question for further research is not only whether compressed workweeks can be implemented with a low risk of adverse health or organizational effects, but also when or how they have negative versus positive effects. We argue that important questions that remains to be examined in future studies are both details of the compressed workweek, such as the length of shift and which alternative work schedules are the comparison, as well as contextual factors when working the compressed week, such as work intensity, work quality and other working conditions.

### Methodological challenges in the included studies

The existing studies on compressed workweeks have some methodological challenges which should be addressed in future studies. In their literature review on compressed workweeks, Bambra et al. (2008) highlighted several methodological challenges in existing studies such as small samples, inadequate control groups, self-reported measures, and short follow-up times (Bambra et al. 2008). In our updated review some of these challenges are still present.

We included ten interventions, however only three of these met the EPHPP’s requirements as a clinical trial. This is in part due to lack of control groups or unclear allocation procedures for intervention and control groups. In line with the studies identified by Bambra et al. (2008), several of the intervention studies suffered from small samples and short follow-up times. Most interventions followed only a few employees from one or a limited number of work places. In fact, two interventions included less than 50 employees and only one intervention included more than 400 employees, and in that intervention extended shifts were only one of multiple changes implemented (Bacon et al. 2005). Among the longitudinal observational studies however, there were studies with substantially larger sample sizes. To address the highly mixed results in current literature future studies should include intervention studies with larger sample sizes, including employees from multiple workplaces.

Most intervention studies had follow-up periods from 6 to 12 months after the intervention. Only two intervention studies had follow-up measures more than one year after the intervention. One of these investigated performance (Barnum 2011) and one satisfaction (Bacon et al. 2005). Among the longitudinal observational studies there were several studies spanning longer timer periods—but most did not investigate long-term effects (> 1 year). There is a clear need for more studies investigating the long-term effects of compressed work schedules.

Noteworthy, none of the studies identifying insignificant relationships used non-inferiority tests (e.g. testing if compressed workweeks are not worse than the alternative, rather than not finding sufficient support for compressed workweeks being wors at a given outcome). Several studies demonstrated insignificant differences between employees working compressed schedules and traditional shorter shifts. Because it is important to know whether compressed workweeks can be implemented without risk to employee health or organizational performance it is important to note that absence of evidence is not the same as evidence of absence (Altman and Bland 1995). Future studies should actively include non-inferiority tests on non-significant findings, or power estimates indicating what effect sizes they are able to detect.

There was also a lack of studies addressing the potential healthy worker effect and the effect of compressed workweek on employees with reduced health. If employees who adverse health effect from working compressed work schedule are more prone to leaving the organization, it would like skew the results. Furthermore, it would have important policy implications as several of the sectors using compressed schedules, such as the police force, often suffer from staffing shortages (Wilson and Grammich 2024).

Finally, despite mixed results in the current and prior literature review, no studies investigated when, for whom, or under which conditions compressed schedules may be beneficial or harmful. Thus, one potential reason for the mixed findings is the wide range of variations in work characteristics in the samples. Arguably, the consequences of working longer hours per day may vary depending on the intensity of the shift, the physical, mental and emotional work demands, number and length of breaks taken, and so forth. From a theoretical standpoint it is argued that strain is not necessarily harmful, but when sustained over a longer period, strain can become harmful (Hunter and Wu 2016; Meijman 1998). Based on this reasoning we expect that the level of strain employees experience at work will be more important when they need to sustain that level for a longer period during the day. Future studies should aim to fill this gap and investigate when and under which conditions compressed schedules may be beneficial or harmful, using work characteristics as moderators.

### Strengths and limitations in the current systematic review

The current review applied a transparent and rigorous systematic method of searching, screening, and quality assessing the studies according to predefined criteria in several electronic databases. Still, there are some limitations to be discussed.

First, there were challenges in distinguishing which studies investigate compressed workweeks when the authors did not specify this clearly, or asked the respondents, how an extended shift is organized. We have thus excluded studies where participants report the length of a usual shift—without control for weekly work hours or days worked a week. The argument is, as stated above, that an extended shift then may be part of a long workweek, and not necessarily a compressed week. However, the extent to which this happens will likely vary across countries and sectors, and for many a compressed workweek is likely to be implied when discussing extended shifts. It is therefore important to recognize that in the pursuit of summarizing studies focusing precisely on compressed workweeks we might have excluded studies on extended shifts, which indirectly captured compressed work schedules. Similarly, we excluded studies focusing on outcomes during, or after, a single shift because they do not take the workweek (or additional days off) into account. By excluding these studies we have likely excluded outcomes generally studied by focusing on single shifts—such as drowsy driving after shift (Scott et al. 2007). It is important to interpret the findings of the present study together with other reviews looking at extended shifts more broadly.

Second, the studies were heterogenous in which and how work schedules and outcomes were operationalized –complicating generalization across studies and excluding the possibility of meta-analyses. For example, the six studies investigating sickness absence were heterogeneous in type of compressed schedule, control group, and operationalizing of absence. In the current review we have used EPHPP to assess the global rating of each study and level of evidence for each outcome. It is important to note that different quality assessment tool may yield different results (Bilotta et al. 2014; Voss and Rehfuess 2013) and using a count approach to synthesizing has clear limitations such as accounting for difference in sample size and handling non-significant findings (see below). Future studies should strive to also replicate findings from prior studies in new populations—comparing similar work schedules and using similar outcomes, thus facilitating the possibility to aggregate findings across populations and countries in meta-analyses.

Thirdly, the results consist of multiple non-significant findings that are difficult to synthesize. In the current review we consider a significant and non-significant result as contradicting. However, if some of these studies are non-significant for reasons such as a small sample size—arguably they are not necessarily contradictory of a significant study. For instance, for two health outcomes, cardiovascular health and quality of life, we have assessed the evidence as mixed based on studies showing reduced cardiovascular health and no significant difference, and improved quality of life and no significant difference.

### Practical implications

Employers and employees may want to implement a compressed workweek for a multitude of reasons. We see a strong growth in compressed schedules in countries such as Norway (Fevang et al. 2024). In other countries researchers and practitioners are advocating for the need to reduce extended shifts (Geiger-Brown and Trinkoff 2010; Harris et al. 2015). Based on the current review, it is unclear whether extended shifts may be used within a compressed schedule without negative consequences for employee health and organizational outcomes. While the current findings are not unequivocally negative, they are also far from endorsing the implementation of compressed workweeks.

So far, it seems relevant for practitioners to consider the type of compressed workweek and what the alternative workhour arrangement is. In the current review, studies comparing compressed workweeks to fixed 8 h day work predominantly reported negative health outcomes, suggesting that a compressed workweek is inadvisable when traditional day work is the alternative. Similarly, all three studies analysing frequency of ≥ 12 h shifts not explicitly organized in a compressed schedule (but controlling for weekly work hours) also supported negative health consequences. The results thus also caution against 12 h shift when they are not fully organized in a compressed schedule. The results for a compressed 10 h shift schedule on the other hand were optimistic, cautiously indicated positive health and performance consequences. However, the evidence was scarce. Finally, evidence for a compressed schedule with 12 h shifts compared to an 8 h shiftwork schedule were mixed for both health and performance, making potential outcomes highly uncertain.

The evidence moderately supports that employees working compressed workweeks (of 10 or 12 h) are more satisfied with their work schedule. Practitioners considering whether to implement or abandon compressed workweekschedules compared to non-compressed shift are thus faced with a difficult dilemma; if a compressed workweekis desired by employees– how certain should they be about the presence or absence of negative outcomes before allowing or denying the use of the schedule?

## Conclusion

The consequences of compressed workweeks are still highly uncertain, with the 20 included studies yielding mixed results for both employee health and performance. The existing longitudinal evidence base of studies cautiously support that a compressed workweek is related to higher sickness absence but also improves satisfaction with shift system. Moreover, the results suggest that the consequence of a compressed schedule is likely dependent on the type of compressed schedule and the alternative schedule. The outcome was predominantly negative when a compressed schedule was compared to a fixed day schedule and when analyzing frequency of 12 h shifts not specifically organized in a compressed schedule. Despite mixed results no studies investigated when or under which conditions compressed workweeks may be beneficial or harmful, though the collective evidence base do suggest that the length of shift and alternative schedule are likely to be of importance.

## References

[CR1] Altman DG, Bland JM (1995) Absence of evidence is not evidence of absence. BMJ 311:485. 10.1136/bmj.311.7003.485 7647644 10.1136/bmj.311.7003.485 PMC2550545

[CR2] Amendola KL, Weisburd D, Hamilton EE, Jones G, Slipka M (2011) An experimental study of compressed work schedules in policing: advantages and disadvantages of various shift lengths. J Exp Criminol 7(4):407–442. 10.1007/s11292-011-9135-7

[CR3] Armijo-Olivo S, Stiles CR, Hagen NA, Biondo PD, Cummings GG (2012) Assessment of study quality for systematic reviews: a comparison of the Cochrane Collaboration Risk of Bias Tool and the Effective Public Health Practice Project Quality Assessment Tool: methodological research. J Eval Clin Pract 18(1):12–18. 10.1111/j.1365-2753.2010.01516.x 20698919 10.1111/j.1365-2753.2010.01516.x

[CR4] Bacon N, Blyton P, Dastmalchian A (2005) The significance of working time arrangements accompanying the introduction of teamworking: Evidence from employees. Br J Ind Relat 43(4):681–701. 10.1111/j.1467-8543.2005.00479.x

[CR5] Bae SH, Fabry D (2014) Assessing the relationships between nurse work hours/overtime and nurse and patient outcomes: systematic literature review. Nurs Outlook 62(2):138–156. 10.1016/j.outlook.2013.10.009 24345613 10.1016/j.outlook.2013.10.009

[CR6] Bambra C, Whitehead M, Sowden A, Akers J, Petticrew M (2008) “A hard day’s night?” The effects of Compressed Working Week interventions on the health and work-life balance of shift workers: a systematic review. J Epidemiol Community Health 62(9):764–777. 10.1136/jech.2007.067249 18701725 10.1136/jech.2007.067249

[CR7] Banakhar M (2017) The impact of 12-hour shifts on nurses’ health, wellbeing, and job satisfaction: a systematic review. J Nurs Educ Pract 7(11):69–83

[CR8] Bannai A, Tamakoshi A (2014a) The association between long working hours and health: a systematic review of epidemiological evidence. Scand J Work Environ Health 40(1):5–18. 10.5271/sjweh.3388 24100465 10.5271/sjweh.3388

[CR9] Bannai A, and Tamakoshi A (2014) The association between long working hours and health: a systematic review of epidemiological evidence. Scand J Work Environ Health 5–18 10.5271/sjweh.3388 24100465

[CR10] Barnum C (2011) Efficiency in Continually Operating Public Organizations: A Case Study. Public Personnel Management, 40(4), 279–292. Retrieved from https://login.ezproxy.hioa.no/login?url=http://search.ebscohost.com/login.aspx?direct=true&db=c8h&AN=108223163&site=ehost-live

[CR11] Battle C, Temblett P (2018a). 12-Hour nursing shifts in critical care: A service evaluation. Journal of the Intensive Care Society, 19(3), 214–218. Retrieved from http://ovidsp.ovid.com/ovidweb.cgi?T=JS&CSC=Y&NEWS=N&PAGE=fulltext&D=emed19&AN=623658195 10.1177/1751143717748094 PMC6110020 30159013

[CR12] Battle C, Temblett P (2018b) 12-Hour nursing shifts in critical care: A service evaluation. The Journal of the Intensive Care Society, 19(3), 214–218. Retrieved from hp://ovidsp.ovid.com/ovidweb.cgi?T=JS&CSC=Y&NEWS=N&PAGE=fulltext&D=prem2&AN=30159013http://openurl.bibsys.no/openurl?sid=OVID:medline&id=pmid:30159013&id=10.1177%2F1751143717748094&issn=1751-1437&isbn=&volume=19&issue=3&spage=214&pages=214-218&date=2018&title=The+Journal+of+the+Intensive+Care+Society&atitle=12-Hour+nursing+shifts+in+critical+care%3A+A+service+evaluation.&aulast=Battle 10.1177/1751143717748094 PMC6110020 30159013

[CR13] Bell LB, Virden TB, Lewis DJ, Cassidy BA (2015) Effects of 13-hour 20-minute work shifts on law enforcement officers’ sleep, cognitive abilities, health, quality of life, and work performance: the phoenix study. Police Q 18(3):293–337. 10.1177/1098611115584910

[CR14] Bernstrøm V, Alves D, Drange I, Houkes I, Nilsen W, Wathne CT (2020) Consequences of compressed work weeks and compressed work-weekends; a systematic review - CRD42020172595. Retrieved from https://www.crd.york.ac.uk/prospero/display_record.php?ID=CRD42020172595 from National Institute for Health and Care Research https://www.crd.york.ac.uk/prospero/display_record.php?ID=CRD42020172595

[CR15] Bilotta GS, Milner AM, Boyd IL (2014) Quality assessment tools for evidence from environmental science. Environ Evid 3:1–14

[CR16] Bonde JPB, Jorgensen Bonzini, Palmer (2013) Risk of miscarriage in relation to work at night, work hours, lifting and standing: A meta-analysis. Occupational and Environmental Medicine. Conference: 23rd Conference on Epidemiology in Occupational Health, EPICOH, 70(no pagination). 10.1136/oemed-2013-101717.213

[CR17] Breslin FC, Dollack J, Mahood Q, Maas ET, Laberge M, Smith PM (2019) Are new workers at elevated risk for work injury? A systematic review. Occup Environ Med 76(9):694–701 31147382 10.1136/oemed-2018-105639

[CR19] Casjens S, Brenscheidt F, Tisch A, Beermann B, Bruning T, Behrens T, Rabstein S (2022a). Social jetlag and sleep debts are altered in different rosters of night shift work. PLoS ONE [Electronic Resource], 17(1), e0262049. Retrieved from https://ovidsp.ovid.com/ovidweb.cgi?T=JS&CSC=Y&NEWS=N&PAGE=fulltext&D=med21&AN=34995309 10.1371/journal.pone.0262049 PMC8740972 34995309

[CR18] Casjens S, Tisch A, Brenscheidt F, Beermann B, Bruening T, Behrens T, Rabstein S (2022b) Investigating the influence of shift work rosters on stress measured as cortisol in hair during the SARS-CoV-2 pandemic. Psychoneuroendocrinology. 10.1016/j.psyneuen.2022.105858 10.1016/j.psyneuen.2022.105858 PMC9251898 35810571

[CR20] Clendon J, Gibbons V (2015a) 12 h shifts and rates of error among nurses: a systematic review. Int J Nurs Stud 52(7):1231–1242. 10.1016/j.ijnurstu.2015.03.011 25910955 10.1016/j.ijnurstu.2015.03.011

[CR21] Clendon J, Gibbons V (2015b) 12 h shifts and rates of error among nurses: a systematic review. Int J Nurs Stud 52(7):1231–1242 25910955 10.1016/j.ijnurstu.2015.03.011

[CR22] Dall’Ora C, Ejebu O-Z, Griffiths P (2022) Because they’re worth it? A discussion paper on the value of 12-h shifts for hospital nursing. Human Resources Health 20(1):36. 10.1186/s12960-022-00731-2 10.1186/s12960-022-00731-2 PMC9077839 35525947

[CR23] Dall’Ora C, Ball J, Recio-Saucedo A, Griffiths P (2016) Characteristics of shift work and their impact on employee performance and wellbeing: a literature review. Int J Nurs Stud 57:12–27. 10.1016/j.ijnurstu.2016.01.007 27045561 10.1016/j.ijnurstu.2016.01.007

[CR24] Dall'Ora C, Ball J, Redfern O, Recio-Saucedo A, Maruotti A, Meredith P, Griffiths P (2019) Are long nursing shifts on hospital wards associated with sickness absence? A longitudinal retrospective observational study. Journal of Nursing Management, 27(1), 19–26. Retrieved from http://ovidsp.ovid.com/ovidweb.cgi?T=JS&CSC=Y&NEWS=N&PAGE=fulltext&D=emexa&AN=626003674 10.1111/jonm.12643 PMC7328726 29978584

[CR25] Desmedt M, Ulenaers D, Grosemans J, Hellings J, Bergs J (2020) Clinical handover and handoff in healthcare: a systematic review of systematic review. Int J Quality Health Care 1–24 10.1093/intqhc/mzaa170 33325520

[CR26] Dionne G, Dostie B (2007). New evidence on the determinants of absenteeism using linked employer-employee data. Industrial & Labor Relations Review, 61(1), 108–120. Retrieved from <Go to ISI>://WOS:000250692500006

[CR27] Driscoll TR, Grunstein RR, Rogers NL (2007) A systematic review of the neurobehavioural and physiological effects of shiftwork systems. Sleep Med Rev 11(3):179–194 17418596 10.1016/j.smrv.2006.11.001

[CR28] Estabrooks C, Cummings G, Olivo S, Squires J, Giblin C, Simpson N (2009) Effects of shift length on quality of patient care and health provider outcomes: systematic review. BMJ Qual Saf 18(3):181–188 10.1136/qshc.2007.024232 19467999

[CR29] Fevang E, Fidjeland A, Gautun H, Lillebråten A, Lillebø OS (2024) Turnusordninger i kommunenes helse-og omsorgstjenester–kraftig vekst i omfang av langvakter, årsturnus og fleksibel turnus. Tidsskrift for Omsorgsforskning 10(3):1–22

[CR30] Ganong WL, Ganong JM, Harrison ET (1976) The 12-hour shift: better quality, lower cost. Journal of Nursing Administration, 17–29 10.1097/00005110-197602000-00009 812964

[CR31] Geiger-Brown J, Trinkoff AM (2010) Is it time to pull the plug on 12-hour shifts?: Part 1. The evidence. JONA J Nurs Administration 40(3):100–102 10.1097/NNA.0b013e3181d0414e 20485206

[CR32] Gracia P, Han W-J, Li J (2021) Nonstandard work schedules in 29 European countries, 2005–15: differences by education, gender, and parental status. Retrieved from https://www.bls.gov/opub/mlr/2021/article/nonstandard-work-schedules-in-29-european-countries-2005-15-differences-by-education-gender-and-parental-status.htm?utm_source=chatgpt.com

[CR33] Griffiths P, Dall'Ora C, Simon M, Ball J, Lindqvist R, Rafferty A-M, Aiken LH (2014). Nurses' shift length and overtime working in 12 European countries: The association with perceived quality of care and patient safety. *Medical Care, 52*(11), 975–981. Retrieved from http://ovidsp.ovid.com/ovidweb.cgi?T=JS&CSC=Y&NEWS=N&PAGE=fulltext&D=psyc11&AN=2014-45337-005 10.1097/MLR.0000000000000233 PMC4196798 25226543

[CR34] Harris R, Sims S, Parr J, Davies N (2015) Impact of 12 h shift patterns in nursing: a scoping review. Int J Nurs Stud 52(2):605–634 25468281 10.1016/j.ijnurstu.2014.10.014

[CR35] Hunter EM, Wu C (2016) Give me a better break: choosing workday break activities to maximize resource recovery. J Appl Psychol 101(2):302–311. 10.1037/apl0000045 26375961 10.1037/apl0000045

[CR36] Imes CC, Barthel NJ, Chasens ER, Dunbar-Jacob J, Engberg SJ, Feeley CA, Baniak L (2023) Shift work organization on nurse injuries: a scoping review. Int J Nurs Stud 138:104395. 10.1016/j.ijnurstu.2022.104395 36481596 10.1016/j.ijnurstu.2022.104395

[CR37] Irvin E, Van Eerd D, Amick BC III, Brewer S (2010) Introduction to special section: systematic reviews for prevention and management of musculoskeletal disorders. J Occup Rehabil 20:123–126 20524048 10.1007/s10926-010-9245-5

[CR38] Kang M-Y, Park H, Seo J-C, Kim D, Lim Y-H, Lim S, Hong Y-C (2012) Long working hours and cardiovascular disease. J Occup Environ Med 54(5):532–537. 10.1097/JOM.0b013e31824fe192 22576460 10.1097/JOM.0b013e31824fe192

[CR39] Kivimaki M, Jokela M, Nyberg ST, Singh-Manoux A, Fransson EI, Alfredsson L, Consortium, I. P.-W. (2015) Long working hours and risk of coronary heart disease and stroke: a systematic review and meta-analysis of published and unpublished data for 603,838 individuals. Lancet 386(10005):1739–1746. 10.1016/S0140-6736(15)60295-1 26298822 10.1016/S0140-6736(15)60295-1

[CR40] Kupperschmidt B (2018) 12 hour shifts: literature reviewed, wise use challenged. J Christ Nurs 35(1):26–32. 10.1097/cnj.0000000000000450 29227388 10.1097/CNJ.0000000000000450

[CR41] Larissa Shamseer DM, Clarke M, Ghersi D, Liberati A, Petticrew M, Shekelle P, Stewart L (2015) Preferred reporting items for systematic review and meta-analysis protocols (PRISMA-P). BMJ 349:7647. 10.1186/2046-4053-4-1 10.1186/2046-4053-4-1 PMC4320440 25554246

[CR42] Leroyer E, Romieu V, Mediouni Z, Becour B, Descatha A (2014) Extended-duration hospital shifts, medical errors and patient mortality. Br J Hosp Med 75(2):96–101 10.12968/hmed.2014.75.2.96 24521805

[CR43] Litwiller B, Snyder LA, Taylor WD, Steele LM (2017) The relationship between sleep and work: a meta-analysis. J Appl Psychol 102(4):682–699. 10.1037/apl0000169 27893255 10.1037/apl0000169

[CR44] Meijman TF, Mulder G (1998) Psychological aspects of workload. In Handbook of work and organi-zational psycholog (Vol. 2, pp. 5–33). Hove,England: Psychology Press

[CR45] Mills MJ, Grotto AR (2017) Who can have it all and how? An empirical examination of gender and work-life considerations among senior executives. Gend Manage 32(2):82–97. 10.1108/gm-01-2016-0011

[CR46] Oh JI, Yim HW (2018) Association between rotating night shift work and metabolic syndrome in Korean workers: differences between 8-hour and 12-hour rotating shift work. Industrial Health, 56(1), 40–48. Retrieved from http://ovidsp.ovid.com/ovidweb.cgi?T=JS&CSC=Y&NEWS=N&PAGE=fulltext&D=emed19&AN=623375405 10.2486/indhealth.2017-0072 PMC5800864 29046489

[CR47] Ooi JWL, Er ATW, Lee WC, Chee HC (2021) The 12-hour shift: radiographers’ perspectives and its applicability during a pandemic. Radiography 27(2):512–518. 10.1016/j.radi.2020.11.007 33243565 10.1016/j.radi.2020.11.007 PMC7685134

[CR48] Palmer KT, Bonzini M, Harris EC, Linaker C, Bonde JP (2013) Work activities and risk of prematurity, low birth weight and pre-eclampsia: an updated review with meta-analysis. Occup Environ Med 70(4):213–222. 10.1136/oemed-2012-101032 23343859 10.1136/oemed-2012-101032 PMC3653070

[CR49] Parkinson J, Arcamone A, Mariani B (2018) A pilot study exploring rehabilitation nurses' perceptions of 12-hour shifts. Nursing, 48(2), 60–65. Retrieved from http://ovidsp.ovid.com/ovidweb.cgi?T=JS&CSC=Y&NEWS=N&PAGE=fulltext&D=emed19&AN=620882284 10.1097/01.NURSE.0000529817.74772.9e 29369281

[CR50] Penso A, Loundou DA, Lehucher-Michel MP, Martin F (2022) Mise au point sur l’effet du travail en 12heures de jour chez le personnel infirmier hospitalier et sur la prise en charge des patients. Arch Malad Profession L’environ 83(6):545–557. 10.1016/j.admp.2022.07.001

[CR51] Persson R, Garde AH, Schibye B, Orbaek P (2006) Building-site camps and extended work hours: A two-week monitoring of self-reported physical exertion, fatigue, and daytime sleepiness. Chronobiology International, 23(6), 1329–1345. Retrieved from http://ovidsp.ovid.com/ovidweb.cgi?T=JS&CSC=Y&NEWS=N&PAGE=fulltext&D=psyc5&AN=2006-23625-022 10.1080/07420520601058021 17190717

[CR52] Puttonen S, Karhula K, Ropponen A, Hakola T, Sallinen M, Harma M (2022) Sleep, sleepiness and need for recovery of industrial employees after a change from an 8- to a 12-hour shift system. Industrial Health, 60(2), 146–153. Retrieved from https://ovidsp.ovid.com/ovidweb.cgi?T=JS&CSC=Y&NEWS=N&PAGE=fulltext&D=med21&AN=34657895 10.2486/indhealth.2021-0052 PMC8980689 34657895

[CR53] Rodriguez Santana I, Anaya Montes M, Chalkley M, Jacobs R, Kowalski T, Suter J (2020) The impact of extending nurse working hours on staff sickness absence: Evidence from a large mental health hospital in England. International Journal of Nursing Studies Vol 112 2020, ArtID 103611, 112. Retrieved from https://ovidsp.ovid.com/ovidweb.cgi?T=JS&CSC=Y&NEWS=N&PAGE=fulltext&D=psyc17&AN=2020-88683-001 10.1016/j.ijnurstu.2020.103611 PMC7700891 32451063

[CR54] Ropponen A, Koskinen A, Puttonen S, Harma M (2020) A case-crossover study of age group differences in objective working-hour characteristics and short sickness absence. Journal of Nursing Management, 07, 07. Retrieved from http://ovidsp.ovid.com/ovidweb.cgi?T=JS&CSC=Y&NEWS=N&PAGE=fulltext&D=medp&AN=32145050 10.1111/jonm.12992 32145050

[CR55] Sallinen M, Kecklund G (2010) Shift work, sleep, and sleepiness—differences between shift schedules and systems. Scandinavian journal of work, environment & health, 121–133. 10.5271/sjweh.2900 20119631

[CR56] Schardt C, Adams MB, Owens T, Keitz S, Fontelo P (2007) Utilization of the PICO framework to improve searching PubMed for clinical questions. BMC Med Inform Decis Mak 7:1–6 17573961 10.1186/1472-6947-7-16 PMC1904193

[CR57] Scott LD, Rogers AE, Hwang W-T, Zhang Y (2006) Effects of critical care nurses' work hours on vigilance and patients' safety. American Journal of Critical Care, 15(1), 30–37. Retrieved from http://ovidsp.ovid.com/ovidweb.cgi?T=JS&CSC=Y&NEWS=N&PAGE=fulltext&D=psyc5&AN=2005-16802-002 16391312

[CR58] Scott LD, Hwang W, Rogers AE, Nysse T, Dean GE, Dinges DF, Dinges DF (2007) The relationship between nurse work schedules, sleep duration, and drowsy driving. Sleep, 30(12), 1801–1807. Retrieved from https://login.ezproxy.hioa.no/login?url=http://search.ebscohost.com/login.aspx?direct=true&db=c8h&AN=105895648&site=ehost-live 10.1093/sleep/30.12.1801 PMC2276124 18246989

[CR59] Shochat T, Hadish-Shogan S, Banin Yosipof M, Recanati A, Tzischinsky O (2019). Burnout, Sleep, and Sleepiness during Day and Night Shifts in Transition from 8- to 12-Hour Shift Rosters among Airline Ground Crew Managers. Clocks & Sleep, 1(2), 226–239. Retrieved from https://ovidsp.ovid.com/ovidweb.cgi?T=JS&CSC=Y&NEWS=N&PAGE=fulltext&D=pmnm4&AN=33089166 10.3390/clockssleep1020020 PMC7445838 33089166

[CR60] Slavin RE (1995) Best evidence synthesis: an intelligent alternative to meta-analysis. J Clin Epidemiol 48(1):9–18 7853053 10.1016/0895-4356(94)00097-a

[CR61] Solovieva S, Lallukka T, Virtanen M, Viikari-Juntura E (2013) Psychosocial factors at work, long work hours, and obesity: a systematic review. Scand J Work Environ Health 39(3):241–258. 10.5271/sjweh.3364 23592217 10.5271/sjweh.3364

[CR62] Su S-B, Lu C-W, Kao Y-Y, Guo H-R (2008. Effects of 12-hour rotating shifts on menstrual cycles of photoelectronic workers in Taiwan. Chronobiology International, 25(2–3), 237–248. Retrieved from http://ovidsp.ovid.com/ovidweb.cgi?T=JS&CSC=Y&NEWS=N&PAGE=fulltext&D=psyc6&AN=2008-07476-007 10.1080/07420520802106884 18533326

[CR63] Surani S, Murphy J, Shah A (2007) Sleepy nurses: are we willing to accept the challenge today? Nursing administration quarterly, 31(2), 146–151. Retrieved from https://login.ezproxy.hioa.no/login?url=http://search.ebscohost.com/login.aspx?direct=true&db=c8h&AN=106287894&site=ehost-live 10.1097/01.NAQ.0000264863.94958.40 17413508

[CR64] Tanaka K, Takahashi M, Hiro H, Kakinuma M, Tanaka M, Kamata N, Miyaoka H (2010) Differences in medical error risk among nurses working two- and three-shift systems at teaching hospitals: a six-month prospective study. Ind Health 48(3):357–364. 10.2486/indhealth.48.357 20562512 10.2486/indhealth.48.357

[CR65] Trinkoff AM, Le R, Geiger-Brown J, Lipscomb J, Lang G (2006) Longitudinal relationship of work hours, mandatory overtime, and on-call to musculoskeletal problems in nurses. Am J Ind Med 49(11):964–971 16691609 10.1002/ajim.20330

[CR66] van Melick MJ, van Beukering MD, Mol BW, Frings-Dresen MH, Hulshof CT (2014) Shift work, long working hours and preterm birth: a systematic review and meta-analysis. Int Arch Occup Environ Health 87(8):835–849. 10.1007/s00420-014-0934-9 24584887 10.1007/s00420-014-0934-9

[CR67] Vila B (2006) Impact of long work hours on police officers and the communities they serve. Am J Ind Med 49(11):972–980. 10.1002/ajim.20333 17006951 10.1002/ajim.20333

[CR68] Virtanen M, Jokela M, Nyberg ST, Madsen IE, Lallukka T, Ahola K, Kivimaki M (2015) Long working hours and alcohol use: systematic review and meta-analysis of published studies and unpublished individual participant data. BMJ 350:g7772. 10.1136/bmj.g7772 25587065 10.1136/bmj.g7772 PMC4293546

[CR69] Voss PH, Rehfuess EA (2013) Quality appraisal in systematic reviews of public health interventions: an empirical study on the impact of choice of tool on meta-analysis. J Epidemiol Community Health 67(1):98–104 22851579 10.1136/jech-2011-200940

[CR70] Wadsworth LL, Facer RL, Arbon CA (2010) Alternative work schedules in local government: cui bono? Rev Public Pers Administr 30(3):322–340. 10.1177/0734371x10368223

[CR71] Wijaya, M. I., Mohamad, A. R., & Hafizurrachman, M. (2020). Shift schedule realignment and patient safety culture. *International journal of health care quality assurance. ahead of print, 15*. Retrieved from ht://ovidsp.ovid.com/ovidweb.cgi?T=JS&CSC=Y&NEWS=N&PAGE=fulltext&D=emexb&AN=630812576 h://openurl.bibsys.no/openurl?sid=OVID:embase&id=pmid:32012498&id=10.1108%2FIJHCQA-04-2019-0080&issn=0952-6862&isbn=&volume=ahead-of-print&issue=aheadofprint&spage=&pages=&date=2020&title=International+journal+of+health+care+quality+assurance&atitle=Shift+schedule+realignment+and+patient+safety+culture&aulast=Wijaya 10.1108/IJHCQA-04-2019-0080 32012498

[CR72] Wilson JM, Grammich CA (2024) Reframing the police staffing challenge: a systems approach to workforce planning and managing workload demand. Policing A J Policy Pract. 10.1093/police/paae005

[CR73] Wong LR, Flynn-Evans E, Ruskin KJ (2018) Fatigue risk management: the impact of anesthesiology residents’ work schedules on job performance and a review of potential countermeasures. Anesth Analg 126(4):1340–1348. 10.1213/ane.0000000000002548 29049076 10.1213/ANE.0000000000002548

